# Downregulation of let-7 by Electrical Acupuncture Increases Protein Synthesis in Mice

**DOI:** 10.3389/fphys.2021.697139

**Published:** 2021-08-20

**Authors:** Ying Huang, Manshu Yu, Akihiro Kuma, Janet D. Klein, Yanhua Wang, Faten Hassounah, Hui Cai, Xiaonan H. Wang

**Affiliations:** ^1^Renal Division, Department of Medicine, Emory University, Atlanta, GA, United States; ^2^Department of Nephrology, The Second Xiangya Hospital of Central South University, Changsha, China; ^3^Renal Division, Affiliated Hospital of Nanjing University of Chinese Medicine, Nanjing, China; ^4^Second Department of Internal Medicine, School of Medicine, University of Occupational and Environmental Health, Kitakyushu, Japan; ^5^Section of Nephrology, Atlanta VA Medical Center, Decatur, GA, United States

**Keywords:** Acu/LFES, exosome, IGF-1 signaling, microRNA, mTOR, skeletal muscle

## Abstract

**Background:**

Our previous study found that acupuncture with low frequency electrical stimulation (Acu/LFES) prevents muscle atrophy by attenuation of protein degradation in mice. The current study examines the impact of Acu/LFES on protein synthesis.

**Method:**

C57/BL6 mice received Acu/LFES treatment on hindlimb for 30 min once. Acu/LFES points were selected by WHO Standard Acupuncture Nomenclature and electric stimulation applied using an SDZ-II Electronic acupuncture instrument. Muscle protein synthesis was measured by the surface-sensing of translation (SUnSET) assay. Exosomes were isolated using serial centrifugation and concentration and size of the collected exosomes were measured using a NanoSight instrument. The mature microRNA library in serum exosomes was validated using a High Sensitivity DNA chip.

**Results:**

Protein synthesis was enhanced in the both hindlimb and forelimb muscles. Blocking exosome secretion with GW4869 decreased the Acu/LFES-induced increases in protein synthesis. MicroRNA-deep sequencing demonstrated that four members of the Let-7 miRNA family were significantly decreased in serum exosomes. Real time qPCR further verified Acu/LFES-mediated decreases of let-7c-5p in serum exosomes and skeletal muscles. In cultured C2C12 myotubes, inhibition of let-7c not only increased protein synthesis, but also enhanced protein abundance of Igf1 and Igf1 receptors. Using a luciferase reporter assay, we demonstrated that let-7 directly inhibits Igf1.

**Conclusion:**

Acu/LFES on hindlimb decreases let-7-5p leading to upregulation of the Igf1 signaling and increasing protein synthesis in both hindlimb and forelimb skeletal muscles. This provides a new understanding of how the electrical acupuncture treatment can positively influence muscle health.

## Highlights

– Protein synthesis is a core physiologic and biological process, occurring inside cells, balancing the loss of cellular proteins through the production of new proteins and degradation of old proteins.
– Proteins perform a number of critical physiologic functions as enzymes, structural proteins or hormones. Protein synthesis plays a key role in many diseases.
– Acupuncture is a form of alternative medicine and a simple and safe treatment. It has been used to treat a wide range of diseases or disorder conditions.
– Our group found that acupuncture plus low frequency electrical stimulation (Acu/LFES) can limit muscle wasting.
– We have evidence that Acu/LFES can increase protein synthesis through decreases in let-7-5p microRNA.
– Since let-7 directly targets and inhibits Igf-1, the decrease in let-7 could lead to Igf1 signaling, which could be the foundation for the rise in protein synthesis.
– Our study provides evidence for a muscle atrophy treatment—a simple method for increasing muscle protein synthesis.

## Introduction

Numerous studies have demonstrated that acupuncture with low frequency electrical stimulation (Acu/LFES) can correct muscle atrophy in human and animals with various diseases, including diabetes and chronic kidney disease-induced muscle wasting (Hu et al., 2015; Su et al., 2015), hindlimb suspension induced muscle loss (Onda et al., 2011), facioscapulohumeral muscle dystrophy (Liu et al., 2019), and amyotrophic lateral sclerosis or sciatic nerve injury caused muscle atrophy (Su et al., 2016; Sudhakaran, 2017; Yu et al., 2017). Acu/LFES is used worldwide as a therapeutic intervention to reduce stress and other health problems (NIH Consensus Conference, 1998). However, little is known regarding the precise mechanisms of this treatment on protein metabolism in muscle.

Skeletal muscle protein metabolism accounts for the major change of the total body protein pool and a fine balance between protein synthesis and protein breakdown regulates skeletal muscle mass (Mitch and Goldberg, 1996). Important determinants of protein synthesis are the key anabolic hormone insulin, insulin-like growth factor 1 (Igf1) and the insulin/Igf1 pathway. Activation of this pathway will upregulate PI3K-Akt-mTOR leading to phosphorylation of mechanistic target of rapamycin complex 1 (mTORC1), and subsequent downstream 4E-binding protein-1 (4EBP1), and the ribosomal protein of 70-kDa S6 kinase 1 (p70S6K1) (Holz et al., 2005). As the name suggests, p70S6K’s target substrate is the S6 ribosomal protein. Phosphorylation of S6 initiates protein synthesis at the ribosome and proliferation of satellite cells results to muscle mass increase (Chung et al., 1992). The phosphorylation of p70S6K at threonine 389 has been used as a hallmark of activation by mTOR. It is well known that resistance exercise stimulates mTORC1 activity, promoting increases in the rates of myofibrillar protein synthesis.

Muscle is recognized as an endocrine organ. Contracting skeletal muscles have the capacity to communicate with other organs through the release of factors, such as myokines and exosomes for intercellular and inter-organ communication (Wang B. et al., 2019; Wang H. et al., 2019). Exosomes are small membranous vesicles that are secreted from muscle fibers inside multivesicular bodies. The release of exosomes is a common cellular function in living biological systems (Kim et al., 2015). The exosomes could act as messengers in tissue crosstalk since these muscle-derived nano-sized vesicles have the ability to deliver useful or harmful molecules (such as cytokines, proteins and miRNAs) to distant organs as well as distant muscles. Both pre-miRNAs and mature miRNAs packaged in exosomes are quite stable (Li et al., 2015). These miRs play an important regulatory role in the mechanisms of adaptation to physiology and pathology conditions. Our previous studies of exosomes following Acu/LEFS found that this treatment alters the expression of multiple miRNAs that are capable of regulating the physiology in distant organs, including decreased let-7 miRNA (Su et al., 2018).

Lethal-7 (let-7) was the second microRNAs (miRNA) to ever be identified. It was originally discovered in the nematode elegans in 2000 (Reinhart et al., 2000). Later, let-7 miRNAs were found in various animal species, including human. Unlike the nematode and fruit fly, which have a single isoform, the let-7 family is composed of nine mature let-7 miRNAs encoded by 12 different genomic loci, some of which are clustered together in the human (Roush and Slack, 2008). Let-7 miRNAs have been the focus of a variety of approaches for therapy and diagnosis. For example, Let-7 has been widely studied in oncogene, cell cycle and immunology fields. Many studies showed that let-7 enhances antitumor responses by directly targeting the high mobility group A2 oncogenes and RAS genes (Johnson et al., 2005; Mayr et al., 2007). Let-7 has been reported to be closely associated with regulation of cell cycle and leads to inhibitor cell proliferation (Johnson et al., 2007). Studies have revealed that let-7 family members act as key regulators for immune response to pathogenic agents in various diseases (Zhi et al., 2017; Gilles and Slack, 2018). Altogether, the let-7 family provides multiple possible strategies for developing approaches of diagnosis markers and therapy.

In this study, we hypothesize that Acu/LFES increases protein synthesis not only in local muscle, but also in distant muscle, through serum-derived exosomes-encapsulated microRNA. For proof of this hypothesis we: (1) measured protein synthesis in the muscle with or without Acu/LFES treatment and found increased synthesis with treatment; (2) measured exosome cargoes after Acu/LFES, and found that four members of the Let-7 family were significantly decreased by Acu/LFES; and (3) tested the impact of let-7 on the Igf1 signaling pathway and protein synthesis and found that Igf1 is the direct target of let-7.

## Results

### Acu/LFES in Hindlimb Significantly Increases Protein Synthesis in Both Gastrocnemius and Triceps Brachii Muscles

**FIGURE 1:**
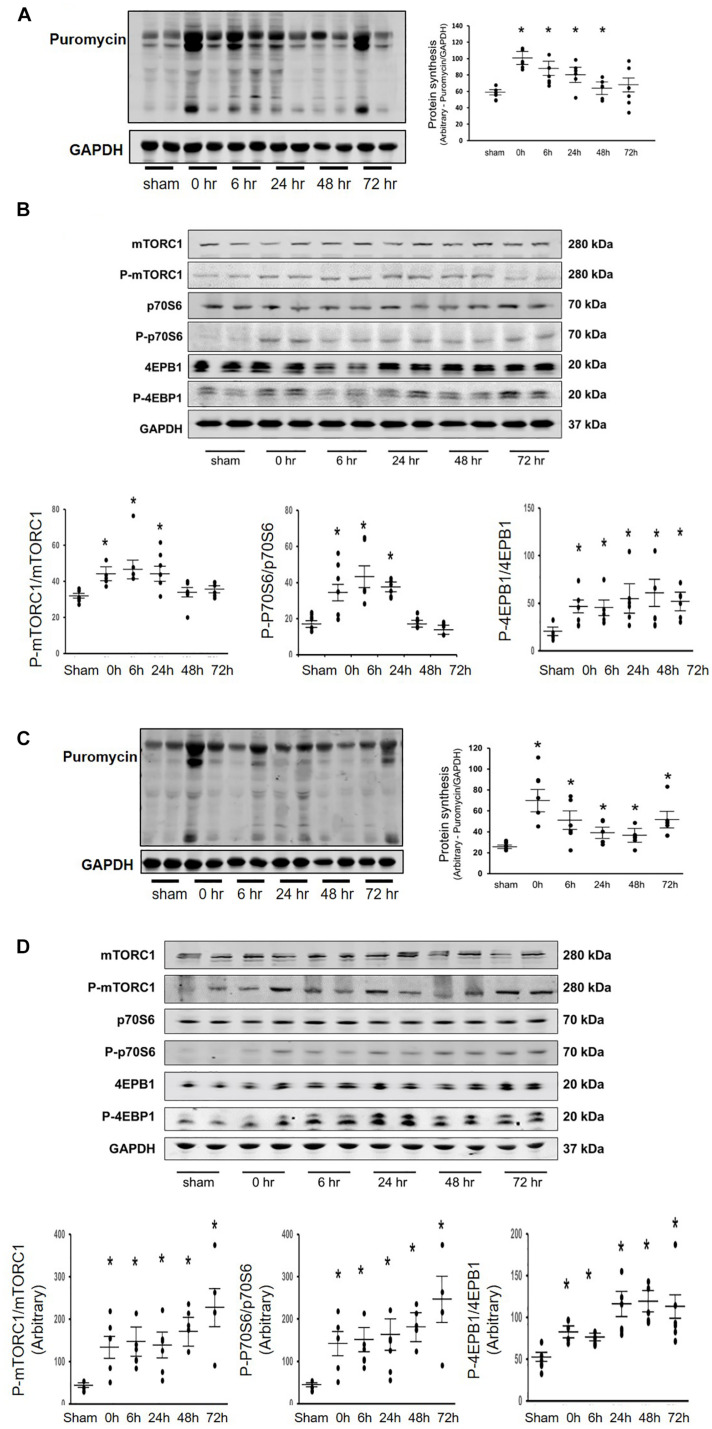
Acu/LFES in hindlimb increased protein synthesis in both gastrocnemius and triceps brachii muscles. Experiments were performed in the sham and Acu/LFES treated mice. Puromycin was injected 30 min before harvest. Protein was isolated from the muscle of mice immediately (0), and 6–, 24–, 48– and 72-h after Acu/LFES. Puromycin in tissue lysates of gastrocnemius muscle **(A)** and triceps brachii muscle **(C)** was measured by Western blots. The point graphs show the change in incorporated puromycin normalized to their corresponding GAPDH protein (*n* = 6/group; *p < 0.05 vs. sham). The proteins mTORC1, 4EBP1 and P70S6 were measured by Western blotting in gastrocnemius muscle **(B)** and triceps brachii muscle **(D)** of sham and Acu/LFES mice. The bottom point graphs show the ratio of phosphorylated protein to total protein, each normalized to the GAPDH from the same sample. Data is provided in arbitrary units (*n* = 6/group; *p < 0.05 vs. sham).

Our previous studies have found that Acu/LFES decreases diabetes-induced protein degradation and improves muscle function (Hu et al., 2015; Su et al., 2015). To investigate whether Acu/LFES alters protein synthesis, we verified the protein synthesis in C57BL/6 mice after Acu/LFES. Acu/LFES mice received needles in point GB34 and S36 of hindlimb using a constant pulse for a one-time 30 min of electrical stimulation. In gastrocnemius muscle, Acu/LFES increased protein synthesis by 1.7-fold immediately after treatment and protein synthesis continued to increase up to the last experimental reading at 48-h (Figure 1A). Protein synthesis signaling proteins phosphorylated mTORC1, p70S6, and 4EBP1 also increased significantly. The increase was apparent immediately (0-h) after treatment through the terminal 24– or 72-h time points in gastrocnemius muscle (Figure 1B). Interestingly, protein synthesis also significantly increased in triceps brachii muscle, which is not near the electrically stimulated area (Figure 1C). These protein synthesis markers increased at 0-h after Acu/LFES until 72 h in triceps brachii muscles (Figure 1D). Acu/LFES did not change the protein degradation markers TRIM63/MuRF1, FBXO32/atrogin-1 and myostatin in these mice (Supplementary Figure 1).

### Blocking Exosome Secretion Limited the Acu/LFES-Induced Increase in Protein Synthesis

**FIGURE 2:**
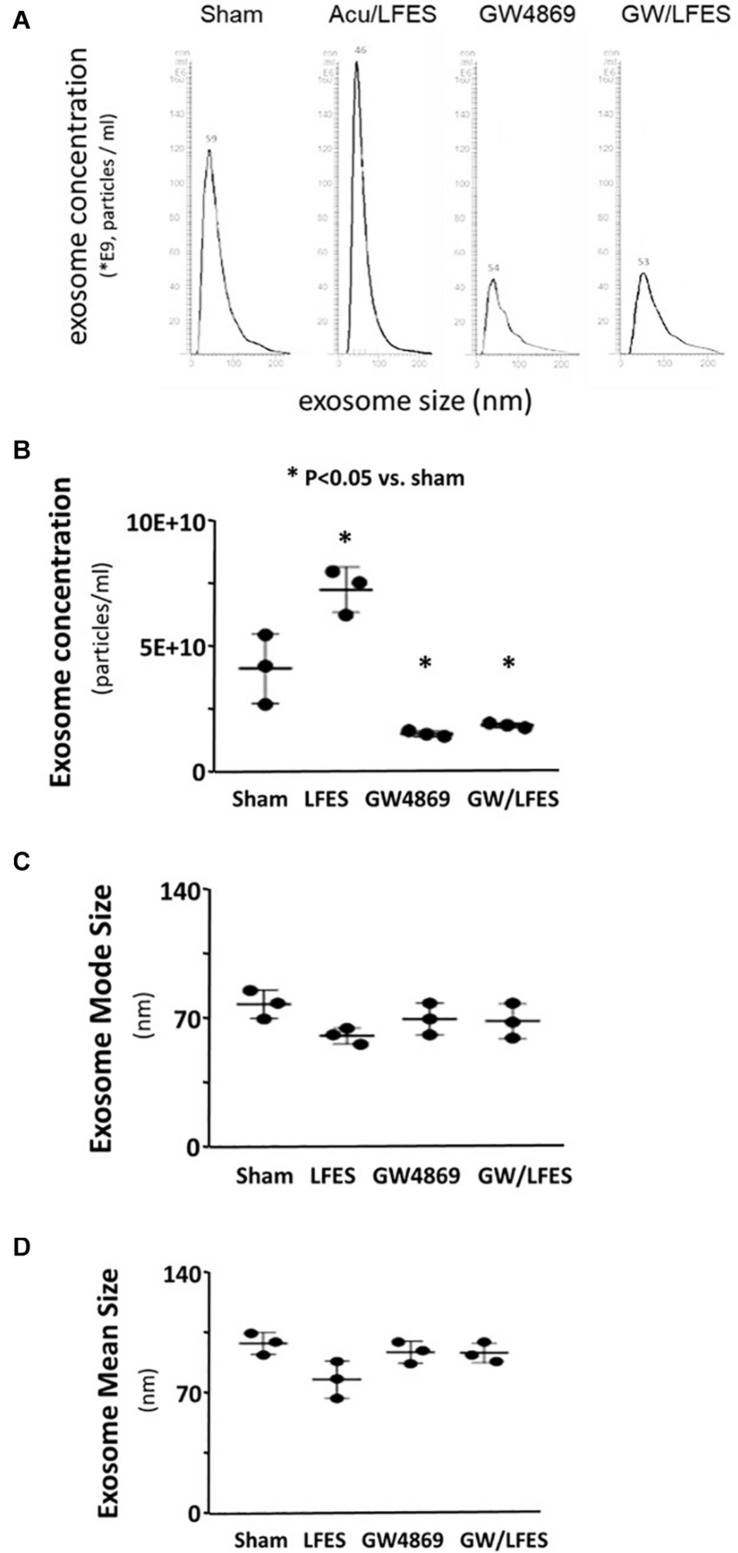
GW4869 limited exosome secretion. Exosomes were isolated from sham, Acu/LFES, GW4869 and Acu/LFES+GW4869 treated mice. The exosome distribution **(A)**, concentration **(B)**, mode size **(C)** and mean size **(D)** were measured using a NanoSight instrument (means ± SE; *n* = 3/group; ^∗^*p* < 0.05 vs. sham).

**FIGURE 3:**
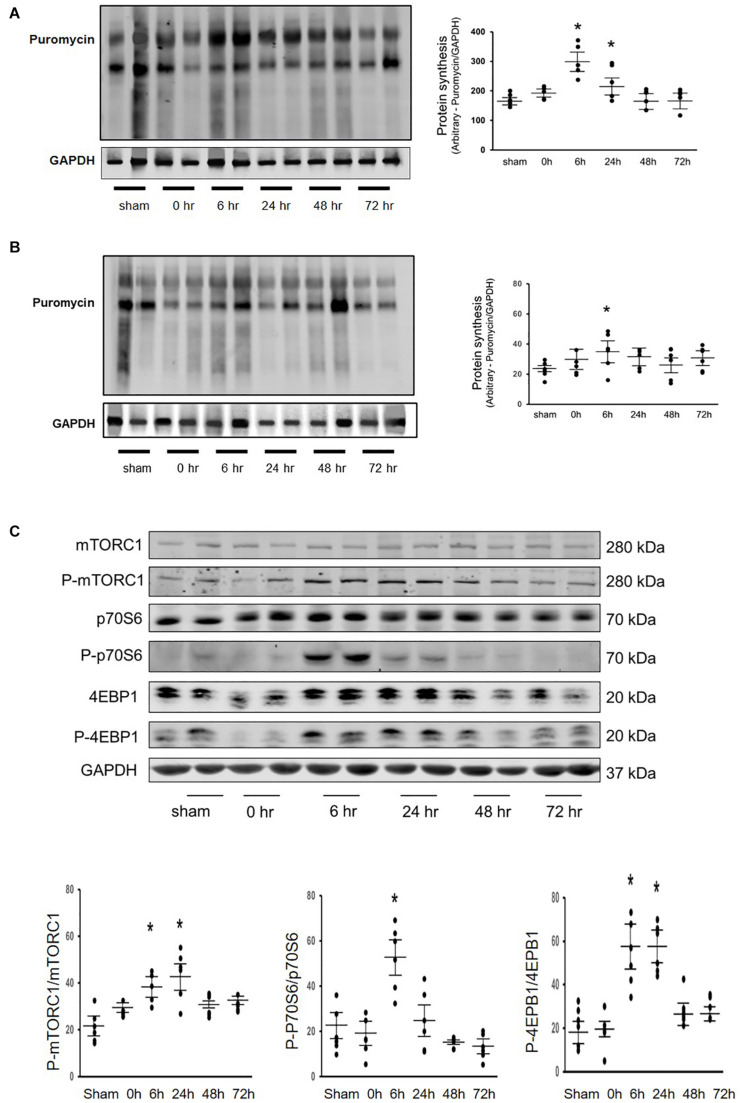
Blocking exosome secretion limited the Acu/LFES-induced increase in protein synthesis. Experiments were performed in the sham and Acu/LFES treated mice. GW4869 was injected 16 h before Acu/LFES and puromycin was injected 30 min before harvest. Protein was isolated from the muscle of mice immediately (0), 6–, 24, 48 and 72 h after Acu/LFES. Puromycin in tissue lysates was measured by Western blots. The point graph of incorporate puromycin into gastrocnemius muscle **(A)** and triceps brachii muscle **(B)** show the change of the density of puromycin protein normalized to their corresponding GAPDH protein (*n* = 6/group; *p < 0.05 vs. sham). The proteins mTORC1, 4EBP1 and P70S6 were measured by Western blotting in gastrocnemius muscle **(C)** of sham and Acu/LFES mice. The bottom point graphs show the ration of phosphate protein to total protein normalized to the same sample GAPDH. Data is provided in arbitrary units (*n* = 6/group; *p < 0.05 vs. sham).

Our previous research found that Acu/LFES increases exosome secretion (Su et al., 2018). To explore whether the increasing protein synthesis in triceps brachii is due to exosome-mediated regulation, we used GW4869 to inhibit exosome secretion. GW4869 is the most widely used pharmacological agent for blocking exosome generation. It inhibits the ceramide-mediated inward budding of multivesicular bodies and prevents release of mature exosomes from multivesicular bodies (Kosaka et al., 2010). Mice were injected with 1 μg/g body weight of GW4869 16-h before Acu/LFES. To elucidate the role of exosomes in Acu/LFES, we isolated exosomes from both sham and Acu/LFES-treated mice with or without GW4869 treatment. A Nanosight instrument was used to measure exosome amounts. We found that Acu/LFES changed exosome distribution in the serum (Figure 2A). The concentration of exosomes was increased 1.7-fold by Acu/LFES (Figure 2B). GW4869 decreased serum exosome concentration by 61% in sham mice, and 73% in Acu/LFES mice. There was no significant difference in either exosome mean size or mode size in each group (Figures 2C,D). In gastrocnemius muscle from mice treated with GW4869, Acu/LFES-induced protein synthesis was apparent at the 6-and 24-h point but not at any later time points (Figure 3A). In triceps brachii muscle, protein synthesis only increased at the 6-h time point after Acu/LFES treatment (Figure 3B). The phosphorylated mTORC1 and 4EBP1 increased at 6– and 24-h time points after treatment, and phosphorylated p70S6 only increased at 6-h′ time point in gastrocnemius muscle (Figure 3C). In triceps brachii muscles p70S6 phosphorylation only increased at the 6-h time point and mTORC1 increased at 0– and 6-h time points (Supplementary Figure 5). These results suggest that Acu/LFES-induced increase in muscle protein synthesis is associated with exosome secretion.

### Acu/LFES Decreased let-7 in Serum Exosomes and Skeletal Muscles

**FIGURE 4:**
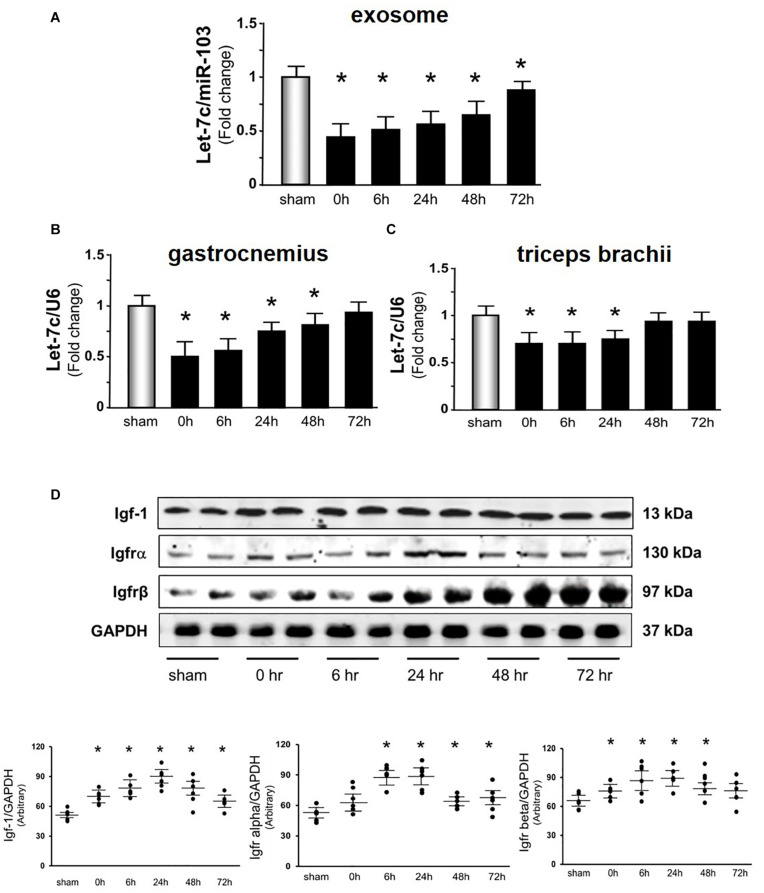
Acu/LFES decreased let-7 in serum exosome and skeletal muscles. Total RNA was isolated from serum exosomes **(A)**, gastrocnemius muscle **(B)** and triceps brachii muscles **(C)** of sham and Acu/LFES treated mice. The expression of let-7c-5p was assayed by real time qPCR. The bar graph shows microRNA from the exosomes of Acu/LFES mice compared with levels in shams (defined as onefold). Results are normalized to miR-103a for serum and U6 for muscle (Bars: mean ± SE.; *n* = 6/group; *p < 0.05 vs. sham). **(D)** Protein was isolated from gastrocnemius muscle. Igf1, Igfrα and Igfrβ measured by Western blots. The point graph of protein showed the change of the density of proteins normalized to their corresponding GAPDH protein Data is provided in arbitrary units. (*n* = 6/group; *p < 0.05 vs. sham).

The Acu/LFES induced increase in protein synthesis in distant muscle could be due to circulating exosomes carrying microRNAs. To explore this possibility, miRNA deep sequencing was performed on serum exosomes from both sham- and Acu/LFES-treated mice. We found that four members of the let-7 miRNA family were significantly decreased by Acu/LFES (Supplementary Figure 2). The largest change was in Let-7c-5p, which was 79% decreased by Acu/LFES treatment. In addition, the expressions of Let-7b-5p, let-7e-5p, let-7a-5p and let-7f-5p were also significantly decreased by 78, 71, 78, and 75% respectively (Table 1). To verify the deep-sequence data, we measured let-7c-5p miRNA by real time qPCR in RNA from serum exosomes of sham and Acu/LFES mice. The expression of let-7c-5p was decreased 75% in the serum exosomes from Acu/LFES mice vs. sham mice (Figure 4A). To validate whether Acu/LFES also altered let-7 miRNA in muscle tissue, the expression of let-7c-5p was measured in skeletal muscle of mice. In gastrocnemius, the expression of let-7c-5p was significantly decreased at 0–, 6–, 24– and 48-h after treatment (Figure 4B). In triceps brachii muscle decrease of let-7 in response to Acu/LFES was apparent at 0–, 6– and 24-h points but not at later time points (Figure 4C).

**TABLE 1:** Let-7s are decreased by Acu/LFES in serum exosome.

**miRNA**	**Base mean**	**Log2 fold change (control/ACU-LFES)**	LfcSE*	Stat**	*p* value***
mmu-let-7c-5p	28,184.6	0.928507	0.218726	4.24507	2.19E-05
mmu-let-7b-5p	18,811.7	0.853183	0.225057	3.79096	0.00015006
mmu-let-7e-5p	480.613	1.01145	0.297435	3.40058	0.00067243
mmu-let-7a-5p	7,705.88	0.640478	0.213673	2.99747	0.00272233
mmu-let-7f-5p	4,335.76	0.590771	0.271134	2.17889	0.0293401
mmu-let-7k	40.8737	0.819582	0.484445	1.6918	0.0906847
mmu-let-7f-5p	4,335.76	0.590771	0.271134	2.17889	0.0293401
mmu-let-7d-3p	2,012.34	0.501061	0.273758	1.83031	0.0672043
mmu-let-7k	40.8737	0.819582	0.484445	1.6918	0.0906847
mmu-let-7d-5p	3,591.96	0.338747	0.229611	1.47531	0.14013
mmu-let-7f-1-3p	3.59731	–0.580753	0.45081	–1.28824	0.197661
mmu-let-7e-3p	17.7148	–0.61289	0.583092	–1.0511	0.293212
mmu-let-7a-1-3p	153.433	–0.368842	0.396564	–0.930093	0.352323
mmu-let-7a-1-3p	153.433	–0.368842	0.396564	–0.930093	0.352323
mmu-let-7c-2-3p	153.433	–0.368842	0.396564	–0.930093	0.352323
mmu-let-7a-2-3p	1.01045	–0.241443	0.324752	–0.743471	0.457197
mmu-let-7f-2-3p	1.05089	–0.243161	0.325405	–0.747256	0.454909
mmu-let-7j	7.3655	–0.38925	0.59186	–0.657673	0.510748
mmu-let-7b-3p	44.5565	0.32332	0.567	0.57023	0.568522
mmu-let-7g-5p	10,722.1	0.148303	0.295198	0.502383	0.615398
mmu-let-7i-3p	5.12321	–0.181665	0.503983	–0.360458	0.718505
mmu-let-7c-1-3p	5.37882	–0.140332	0.513847	–0.273101	0.784776
mmu-let-7i-5p	9,099.88	–0.116069	0.257261	–0.45117	0.865412
mmu-let-7g-3p	0	0	0	0	0

*LfcSE is the standard error of log2 fold change estimate. **Stat = Wald statistic. ***p value = Wald test.

According to a miRNA database search, let-7-5p targets insulin-like growth factor 1, and Igf1 receptor (Igf1r) (Lewis et al., 2005). Since microRNA inhibits protein translation of their targets mRNA, a decrease of let-7 could release the inhibition of protein translation and results to increase targeted proteins, Igf-1 in this case. To identify whether Acu/LFES alters the translation of Igf-1, the amount of Igf1 and Igf1 receptor α and β subunits were measured by western blot (Figure 4D). Igf-1 was significantly increased immediately after Acu/LFES and persisted to the 72-h point. The increase in Igf-1r α subunit was detected from 6– to 72-h, and β subunit was from 0– to 48-h after Acu/LFES.

### Overexpressing let-7c-5p Decreased Protein Synthesis in Cultured C2C12 Myotubes

**FIGURE 5:**
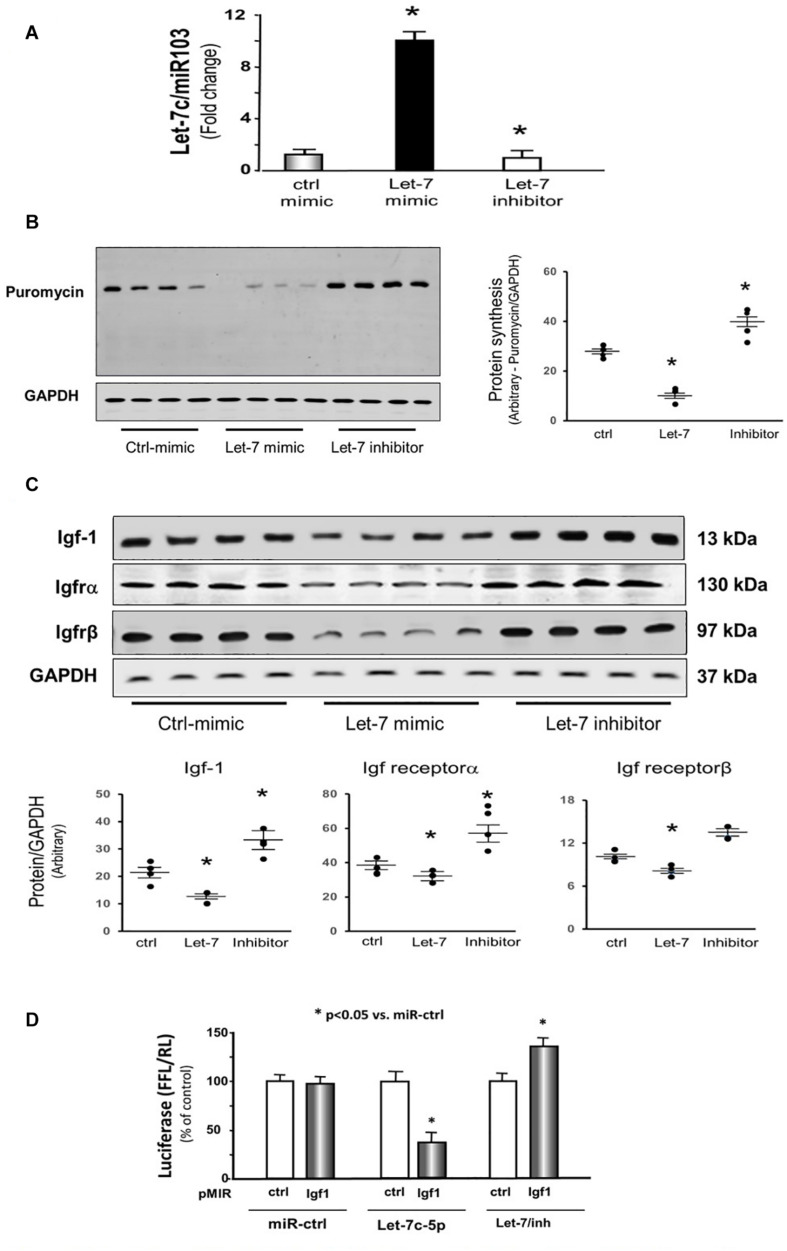
Provision of let-7 inhibited Igf-1, Igfrα and Igfrβ in cultured C2C12 myotubes. **(A)** Total RNA was isolated from C2C12 myotubes transfected with control/mimic, let-7-5p/mimic and let-7/inhibitor. The expression of let-7-5p was assayed by real time qPCR. The bar graph shows let-7-5p from different group compared with levels in control/mimic (defined as onefold). Results are normalized to miR103 (Bars: mean ± SE; *n* = 4/group; *p < 0.05 vs. control/mimic). **(B)** Protein was isolated from the C2C12 myotubes transfected with control/mimic, let-7-5p/mimic and let-7/inhibitor. Puromycin was added into cell culture medium (1 μg final concentration) exactly 30 min before harvesting the cells. Puromycin in cell lysates was measured by Western blots. The point graph of incorporate puromycin into myotube shows the change of the density of puromycin protein normalized to their corresponding GAPDH protein (*n* = 4/group; *p < 0.05 vs. sham). **(C)** Protein was isolated from the C2C12 myotubes transfected with control/mimic, let-7-5p/mimic and let-7/inhibitor. Igf-1, Igfrα and Igfrβ in myotube lysates was measured by Western blots. The point graph showed the change of the density of each protein normalized to their corresponding GAPDH protein. Data is provided in arbitrary units. (*n* = 6/group; *p < 0.05 vs. control/mimic). **(D)** C2C12 cells were transfected with luciferase pMIR–ctrl vector (white bars) or the vector containing the 3′-UTR of Igf1 (pMIR-Igf: black bars). Cells were co-transfected with control mimic (miR-ctrl), let-7c-5p or let-7 inhibitor. Luciferase activity in cells that received the pMIR–ctrl with miR-ctrl was designated as the 100% (far left white bar). The other bars show the response to let-7 expressed as a percent of this control. Triplicate determinations were made in each condition and each experiment was repeated twice; the firefly luciferase (FFL) results were normalized by renilla luciferase (RL) activity. Data: mean ± SE; *n* = 9; * = *p* < 0.05 vs. pMIR-ctrl + miR-ctrl.

To examine whether let-7 could change protein synthesis, we transfected let-7c-5p miRNA mimic or its inhibitor into cultured C2C12 cells. First, we tested whether let-7c-5p was successfully overexpressed. The expression of let-7 was increased 10.2-fold in the cells transfected with let-7c-5p mimic, and 15% decreased following transfection of the let-7 inhibitor compared with the control mimic group (Figure 5A). Second, we measured protein synthesis using puromycin incorporation and found that overexpressing let-7 significantly decreased protein synthesis. Inhibition of endogenous let-7c-5p increased protein synthesis 1.28-fold (Figure 5B).

### Provision of let-7 Inhibited Insulin/Igf-1 Signaling Pathway Components in Cultured C2C12 Myotubes

Since decreasing let-7-5p showed upregulation of insulin/Igf1 signaling pathway in animals, the protein and mRNA levels of these targets were investigated in the cultured cells. Providing let-7c-5p to the C2C12 myotubes in culture resulted in a decline in the protein abundance of Igf1, Igf1r α and β subunits (Figure 5C). Conversely, decreasing endogenous let-7 expression by transfection of the let-7 inhibitor raised the amount of Igf1 and Igf receptorα (Figure 5C). The 3′-UTR of Igf1 contains a conserved binding site for let-7 miRNA according to a consensus sequence search. To experimentally confirm that let-7c-5p directly interacts with the Igf1 mRNA. The Igf1 target site of let-7 (1,360–1,367 nt on Igf1 3′-UTR) was cloned into a luciferase reporter construct (pLUC-Igf1/3UTR). When cells were transfected with pLUC-Igf1/3UTR along with let-7c-5p miRNA, luciferase activity was decreased. However, when cells were transduced with an Igf1-luciferase construct along with let-7c inhibitor, luciferase activity was enhanced (Figure 5D). These results confirmed that let-7-5p directly targets Igf1 and blocks its translation.

## Discussion

In this study, we provide evidence showing that Acu/LFES administered to the hind limb muscles results in enhanced protein synthesis in both hindlimb and forelimb of mice. In addition, we showed that the treatment decreases circulation of let-7 miRNA in exosomes, which has the potential to influence distant muscles. A decrease in let-7 would increase the production of Igf-1 and Igf-1r; therefore, the consequence of let-7 inhibition would be increased protein synthesis.

It is well known that resistance exercise increases muscle mass through upregulation of the IGF-1 signaling pathway. Many tissues secrete Igf-1, including liver and skeletal muscle. Circulating Igf1 is largely contributed by the liver. However, skeletal muscle protein synthesis does not depend on plasma Igf1 (Sjogren et al., 1999). Instead, intrinsic secretion of muscle Igf-1 is a key determinant for activation of protein synthesis in muscle. Activation of the Igf-1 pathway results in phosphorylation of Akt, followed by upregulation of the mechanistic target of rapamycin complex 1 (mTORC1) (Morton et al., 2016). In our study, we found that Acu/LFES mimics resistance exercise, in that it upregulates the Igf-1 signaling pathway for at least 48 h leading to increased protein synthesis. The half-life of IGF-1 is only 5–10 min (Morimoto et al., 2005), so how is the increased protein synthesis supported for 48–72 h? We believe the key is the Acu/LFES -induced decrease in let-7 (Figure 4A) that results in increased levels of Igf-1 for 48–72 h and the consequent activation of protein synthesis (Figure 1A). The lower level of circulating let-7 would remove a repressive effect resulting in activation of Igf-1/Akt/mTORC1 signaling in distant muscles. In addition, the mTOR-dependent protein synthesis pathway is upregulated in 24-h, but increased protein synthesis persists for up to 72-h. We don’t have an explanation for this phenomenon. Possibilities are that the Acu/LFES upregulation of protein synthesis levels are related to an mTOR-independent regulation. Alternatively, they may reflect a process that is begun by increasing mTOR that remains in effect beyond the initial stimulation. This is suggested by the fact that the protein synthesis levels, while significantly elevated, are not at as high a level as when mTOR is activated. Unraveling how mTOR signaling contributes to the protein synthesis during Acu/LFES needs further study. The important point is that patients with severe diseases that have muscle wasting frequently are unable to exercise to stimulate protein synthesis so it is important to explore other treatments that will provide them with a similar benefit. Acu/LFES will help them to increase muscle protein synthesis and prevent muscle wasting.

The let-7 family is involved with maintenance of muscle mass. An increase in let-7 is often associated with muscle loss. Oculopharyngeal muscle dystrophy patients have significantly increased expression of let-7 (Cappelletti et al., 2019). Muscle biopsy studies found that the expression of let-7s was increased in the skeletal muscle in humans with lower limb immobilization (D’Souza et al., 2018). However, there is some controversy about this role. In healthy men with 21-days bed rest let-7 was increased (Rullman et al., 2016); but in another study, the expression of let-7 was decreased in skeletal muscle at 10-days of bed rest in healthy man (Rezen et al., 2014). Our current study found that four members of the let-7 family were decreased in Acu/LFES mice. These changes were associated with an increase in protein synthesis and upregulation of the Igf-1 signaling pathway.

The impact of let-7 on the Igf1 signaling pathway could differ in various tissues. Our current study indicates that let-7c-5p directly targets Igf1 and inhibits its translation in skeletal muscle. The consequence of inhibiting Igf1 is inhibition of the downstream signaling pathway. For example, decreasing the activity or availability of the signaling pathway component mTORC1 would suppress protein synthesis, which is what we see in our Acu/LFESmice. The mTORC1 complex is a key player in nutrient status, and when activated, mTORC1 promotes protein synthesis, lipogenesis, and energy metabolism (Laplante and Sabatini, 2012). Some investigators have demonstrated that let-7 represses mTOR activation without turning off the insulin-signaling pathway in brain (Dubinsky et al., 2014). However, other recent articles describe let-7 directly targeting Igf-1 and/or Igf-1 receptor in human colorectal cancer cell proliferation (Samadi et al., 2019) in endometrial stromal cells (Ghazal et al., 2015) and in cultured testicular fragments (Shen et al., 2014). Another study showed that elevated let-7 expression increased insulin resistance while inhibition of the let-7 reduced insulin resistance and improved glucose uptake in the diabetic myocardium through Akt and mTOR pathways (Li et al., 2016). In our hands, Acu/LFES-induced decreased let-7 at 48 h, but increased Igf-1 for up to 72-h. This could suggest that Acu/LFES-mediated upregulation of Igf-1 is not only dependent on let-7 but has multiple regulatory pathways. This needs further investigation. Acu/LFES uses low-voltage electric currents in acupuncture points to treat a wide range of conditions. We used Acu/LFES for treat CKD-induced muscle wasting. Several alternate electric stimulations have also been used in clinical settings to treat different diseases, including muscle atrophy (Doucet et al., 2012). Transcutaneous electrical nerve stimulation (TENS) therapy involves the use of low-voltage electric currents to treat pain (Gibson et al., 2017). Neuromuscular electrical stimulation (NMES) was used to build muscle strength after surgery or a period of disuse (Doucet et al., 2012). However, these treatments are different from Acu/LFES. Both TENS and NMES are non-invasive while Acu/LFES employs a direct insertion of a needle into the muscle. They also have different targets. TENS is specifically targeting the sensory nerves, which are responsible for sending pain-stop signals to the brain. NMES targets the muscle itself, specifically through the motor nerves to improve muscle regeneration. Acu/LFES targets acupuncture points (explained in the next paragraph). Some investigators performed NMES in hemodialysis patients and found that NMES increased quadriceps muscle area and maximum quadriceps extension strength compared with control patients (Esteve et al., 2017). However, there has been no study that directly compares Acu/LFES with NMES, and there is no study that examines the impact of the NMES on exosome release or exosome carried microRNA. Therefore, we cannot exclude the possibility that external electrical stimulation such as NMES may have beneficial effects similar as Acu/LFES. In this work we provide evidence that Acu/LFES can improve muscle health and may offer therapeutic options in the clinic.

Electrical Acupuncture involves the insertion of very thin needles through the skin at strategic points on the body; it is part of the ancient practice of Traditional Chinese Medicine. The most common role of acupuncture is to treat pain, for stress management and for overall wellness. Traditional Chinese Medicine practitioners believe the human body has more than 2,000 acupuncture points connected by channels or meridians, named Jing-Luo (collaterals). These channels create an energy flow (Qi, pronounced “chee,” which means energy flow) through the body that is responsible for overall health. Disruption of the energy flow can cause disease. By applying Acu/LFES to certain points, it is thought to improve the flow of Qi, thereby improving health for distant organs. However, no anatomy study has identified structural components of the Jing-Luo in the human body. In this study, we found that electrical acupuncture changes the exosome concentration, size, and cargo, such as microRNA, that is in the circulation. We believe that muscle-derived exosomes in circulation play a role in Jing-Luo and exosome-carried cargo plays a role in Qi. This is a new concept to explain ancient medicine.

A consideration in this study is that we used GW4869 to inhibit exosome secretion, since it is the most widely used pharmacological agent for blocking exosome generation. However, GW4869 inhibits sphingolipid metabolism. Sphingolipids are capable of modulating multiple cell functions, such as apoptosis, cell proliferation, differentiation, and inflammation and have a broad role in skeletal muscle (Tan-Chen et al., 2020). Therefore, the potential of the Acu/LFES to influence muscle that is distant from the Acu/LFES origin site through a non-exosome mechanism needs further study.

### Conclusion

Acu/LFES in the hindlimb releases exosomes into the circulation where it could move to and influence distant muscle and increase protein synthesis. The increase in protein synthesis in response to Acu/LFES is a consequence of the decrease in exosome-carried let-7-5p miRNA. Since let-7 targets and inhibits Igf-1 signaling pathway, limitation of let-7 upregulates this pathway and leads to increased protein synthesis. Our study provides strong mechanistic insight for understanding the benefits of treating muscle atrophy with Acu/LFES.

## Materials and Methods

### Animals

These experiments were approved by the Emory University IACUC (protocol 4000152). The mice (C57BL/6J) were purchased from Jackson Laboratories (Bar Harbor, ME, United States) and were housed with a 12-h light/12-h dark cycle. GW4869, an inhibitor of exosome release, was purchased from Sigma-Aldrich. GW4869 was initially dissolved in DMSO into a stock solution of 200 mM before dilution in PBS to 20 μM for final injection and final DMSO concentration is 0.01%. The impact of vehicle (0.01% DMSO) on exosome secretion has also be tested in mice (Supplementary Figure 4).

### Acu-LFES Treatment

The mice were kept in specially designed restraints so that they would remain in a recumbent position during Acu-LFES treatment. Mice were awake without any anesthesia and appeared to be comfortable throughout the treatments. Electrical acupuncture points were according to the WHO Standard Acupuncture guidelines (Lim, 2010). The positive point (anode: Yang-Ling-Quan, GB34) is in the hollow of the exterior-inferior of the caput fibulae about 6 mm deep. This position is close to the superficial fibular nerve and deep fibular nerve. The negative point (cathode: Zu-San-Li, ST36) is 5 mm beneath the capitulum fibulae and located laterally and posterior to the knee-joint about 7 mm deep and close to fibular nerve. The impulses were delivered between the two electrical acupuncture needles. Disposable sterile needles with a diameter of 0.25 mm (Shen Li Medical and Health Material Co., Ltd., Wujiang, China) were used. The needles were connected into an SDZ-II Electronic acupuncture instrument using consistent pulse, electric frequency 20 Hz, electric current 1 mA for 30 min (Hu et al., 2015). Sham mice had Acu/LFES needles inserted in close proximity to the ACU/LFES insertion position, needles were connected to the LFES device, but electrical stimulation was not applied.

### Determination of Protein Synthesis by Puromycin Incorporation

To determine the rate of protein synthesis we utilized surface sensing of translation (SUnSET) methodology (Goodman et al., 2011). *In vivo*, 0.04 μmol/g puromycin (Calibiochem, Catalog #: 540222) was injected intraperitoneally into mice 30 min before harvest of skeletal muscle. Muscle was harvested at 0, 6, 24, 48 and 72 h after treatment and homogenized in Mueller’s Buffer (50 mM HEPES, 0.1% Triton-X100, 4 mM EGTA, 10 mM EDTA, 15 mM Na4P2O7, 100 mM β-glycerophosphate, 20 mM NaF, 5 mM NaVO4 and 1% protease inhibitor cocktail). *In vitro*, C2C12 myotubes were grown in 6-well plates. Puromycin was added into the cell culture medium (1 μM final concentration) exactly 30 min before harvesting the cells. Cells were scraped into ice-cold RIPA buffer (100 μl for one well of a 6-well plate) followed by ultrasound sonication on ice. Puromycin-containing proteins were analyzed by Western blot. Proteins were separated on 10% SDS-PAGE gels. Anti-puromycin antibody was purchased from Millipore (Catalog #: MABE343; Burlington, MA, United States).

### Western Blot and Antibodies

Skeletal muscle or cells were homogenized in Gentle Lysis Buffer (10 mM Tris–HCl, 10 mM NaCl, 2 mM EDTA, 0.5% NP-40, 1% glycerol, and fresh added: 1 mM Na3VO4; 10 μg/ml PMSF; 5 μg/ml Aprotinin; 1 μg/ml Leupeptin) with phosphatase inhibitors cocktail 1 and 2 (Sigma). Protein concentration was measured using a RC-PC protein assay kit (Bio-Rad). Equal amounts of protein were loaded on the acrylamide/bis SDS-PAGE gel. Protein was transferred to a PVDF membrane and blotted with a specific primary antibody. Primary antibodies: (1:1,000 dilution except where indicated) that we used included mTOR (cat# 2972), p-mTOR (Ser2448; cat# 2481), 70S6K (cat# 9202); p-p70S6K (Thr389; cat# 9205), 4E-BP1 (53H11; cat# 9644); phospho-4E-BP1 (Thr37/46; Cat# 2855) from Cell Signaling; GAPDH (SC-365062), igfra (SC-712) and igfrb (SC-713) from Santa Cruz). Anti-IGF1 antibody (ab9572) was purchased from Abcam). Protein bands were scanned and quantified using the Li-cor Odyssey infrared scanning system (Li-COR Biosciences, Lincoln, Nebraska).

### Isolation of Exosome

Exosomes were harvested from mice serum or conditional medium of cultured C2C12 myotubes. Mice sera were obtained from heart puncture. 0.5 ml serum from each mouse was diluted 5-times with PBS before isolation of exosomes. For purification and characterization of exosomes from serum or conditional medium, cell debris and organelles were eliminated by centrifugation at 1,000 *g* for 10 min, 4°C. The supernatant fraction was further centrifuged at 16,000 *g* for 30 min. The second supernatant was sterile filtered through a 0.22 μm filter. Exosomes were pelleted at 120,000 *g* for 90 min at 4°C (L8-70M ultracentrifuge, Beckman-Coulter, Indianapolis, IN, United States). Finally, the exosome pellet was re-suspended in 100-400 μl RNA stabilization reagent (Qiagen, Germantown, MD, United States) for RNA extraction or sterile PBS for other experiments. Exosomal size and concentration were verified using a NanoSight instrument (Malvern, Westborough, MA, United States) and an exosome marker (TSG101) was assessed by Western blot (Su et al., 2018) and exosome images taken by electro-microscopy (Supplementary Figure 3). Exosomes were also isolated from serum of vehicle (0.01% DMSO) injected mice. There are no significant differences in exosome size and concentration between vehicle injected and non-injected mice (Supplementary Figure 4).

### Reverse Transcription and Quantitative PCR (q-PCR) for MicroRNA and mRNA

Total RNA was extracted using Tri-Reagent (Molecular Research Inc., Cincinnati, OH, United States). For miRNA, the miRCURY LNA^TM^ Universal cDNA Synthesis kit (Exiqon Inc., Woburn, MA, United States) was used for reverse transcription of miRNA. The primers were purchased from Exiqon. The miRCURY LNA microRNA PCR SYBR Green master mix (Exiqon Inc.) was used for qPCR with the following cycle parameters: 95°C for 10 min and 40 cycles at 95°C for 10 s and 60°C for 60 s. Expression of individual microRNA was standardized to the mouse U6 gene (tissue) or miR103 (serum) (Hu et al., 2015; Su et al., 2017). For mRNA we used a Thermoscript RT-PCR kit (Invitrogen, Carlsbad, CA, United States). Real-time qPCR was performed with SYBR Green PCR Reagents (Bio-Rad, Hercules, CA, United States) using the following cycle parameters: 94°C for 2 min and 40 cycles at 94°C for 15 s, 55°C for 30 s, 72°C for 30 s with final extension at 72°C for 10 min. The quantification cycle (Cq) values were defined as the number of cycles required for the fluorescence signal to exceed the detection threshold. Individual miRNA or mRNA expression was calculated as the difference between the threshold values of the two genes (2-Δcq). Melting curve analysis was routinely performed to verify the specificity of the reaction. Let-7-5p (YP00204767) was ordered from Qiagen (Germantown, MD, United States).

### miRNA-Seq Library Preparation and Sequencing

Qualitative and quantitative analysis of the total RNA was performed using the Thermo Nanodrop 2000 and Agilent 2100 Bioanalyzer respectively. Small RNA libraries were prepared using the SeqMatic tailormix miRNA sample preparation kit (SeqMatic. Union City, CA, United States) as per manufacturer’s instructions. Briefly, 100 ng of total RNA was used for library preparation. Small RNA’s were ligated with Illumina compatible adapters and each sample was tagged with a unique barcode to allow multiplexing. The adapter-ligated libraries were then enriched using PCR amplification followed by gel enrichment for mature miRNA library. The amplified library was validated using a High Sensitivity DNA chip on the Agilent Bioanalyzer. The libraries were further quantified on Qubit^®^ 2.0 Fluorometer (Life Technologies, Grand Island, NY, United States) using the High Sensitivity dsDNA assay. Libraries from all the samples were multiplexed and run in a single lane of Illumina 3K flowcell. PhiX was used as an internal control on each lane to monitor the error statistics. Cluster generation was performed on the v3 flowcell on the Illumina cBot. The clustered flowcell was sequenced on the Illumina HiSeq3000 system as a 100-cycle single read multiplexed run.

### Cell Culture

C2C12 cells (ATCC, Manassas, VA, United States), studied between passages 3 and 9, were cultured in growth medium (Dulbecco’s Modified Eagle’s Medium (DMEM) with 10% fetal bovine serum, 10% cow serum, 25 mM glucose, 100 u/ml penicillin, 100 μg/ml streptomycin, and 2 mM L-glutamine). Myotube differentiation was induced by replacing growth medium with differentiation media (FBS and cow serum was replaced by 2% horse serum). For *in vitro* let-7 overexpression and inhibition, mmu-let-7c-5p mimic (miRBase Accession #: MIMAT0000523; cat: 4464066), mmu-let-7c-5p inhibitor (Catalog #: 4464084), miRNA mimic negative control #1 and miRNA inhibitor negative control #1 were purchased from Thermo Fisher Scientific (Waltham, MA, United States). For transfection, C2C12 cells in growth medium were seeded in 6-well plates and transfected using Effectene transfection reagent (Qiagen, Valencia, CA, United States). Cells were harvested 48 h after transfection and assayed for microRNA, mRNA and protein.

### Luciferase Reporter Assay and Transfection

Effectene transfection reagent was used for transfection (Qiagen). Renilla luciferase vector was used for transfection efficiency control. Firefly and Renilla luciferase activities were measured by dual-luciferase assays (Promega) using TD-20/20 Luminometer (Turner designs, Sunnyvale, CA, United States) (Du et al., 2014). The luciferase report vectors (pMIR-REPORT Luciferase) were purchase from Applied BIOSYSTEMS (Cat#: AM5795; Waltham, MA, United States). The construct was made by Emory Integrated Genomics Core. Since the Let-7c miRNA binding site on the 3′-UTR of Igf1 is located at 1,360–1,367, the custom-made vector (pLuc-Igf1-3 UTR) containing the firefly luciferase gene and the 3-UTR (1310-1417) of Igf1. The insert was cloned between the *Spe*I and *Hin*dIII of the multiple cloning sites.

### Statistical Analysis

Data are presented as mean ± se. To identify significant differences between two groups, comparisons were made by using the Student’s *t*-test. When multiple treatments were compared, ANOVA was performed with a *post hoc* analysis by the Student–Newman–Keuls test. Differences with *P* values < 0.05 were are considered significant.

## Data Availability Statement

The datasets presented in this study can be found in online repositories. The names of the repository/repositories and accession number(s) can be found below: https://www.ncbi.nlm.nih.gov/, GSE176530.

## Ethics Statement

The animal study was reviewed and approved by Emory University IACUC.

## Author Contributions

XW, CH, YH, and MY: conceptualization. CH, MY, and YH: methodology. XW, YH, MH, YW, and FH: validation. YH and MY: formal analysis. YH, MY, AK, HC, YW, and FH: investigation. XW and HC: resources. XW, YH, and MY: data curation. YH and MY: writing—original draft preparation. XW and JK: writing—review and editing. XW and HC: visualization and funding acquisition. XW: supervision. All authors contributed to the article and approved the submitted version.

## Author Disclaimer

The content is solely the responsibility of the authors and does not necessarily reflect the official views of the NIH, the Department of Veterans Affairs, or the United States Government.

## Conflict of Interest

The authors declare that the research was conducted in the absence of any commercial or financial relationships that could be construed as a potential conflict of interest.

## Publisher’s Note

All claims expressed in this article are solely those of the authors and do not necessarily represent those of their affiliated organizations, or those of the publisher, the editors and the reviewers. Any product that may be evaluated in this article, or claim that may be made by its manufacturer, is not guaranteed or endorsed by the publisher.
