# Supplementary material for: Downregulation of let-7 by Electrical Acupuncture Increases Protein Synthesis in Mice
Source: Front Physiol. 2021 Aug 20;12:697139. doi: 10.3389/fphys.2021.697139 (PMC8417904; doi:10.3389/fphys.2021.697139)
Supplement: Supplementary file 1 [file Data_Sheet_1.pdf]

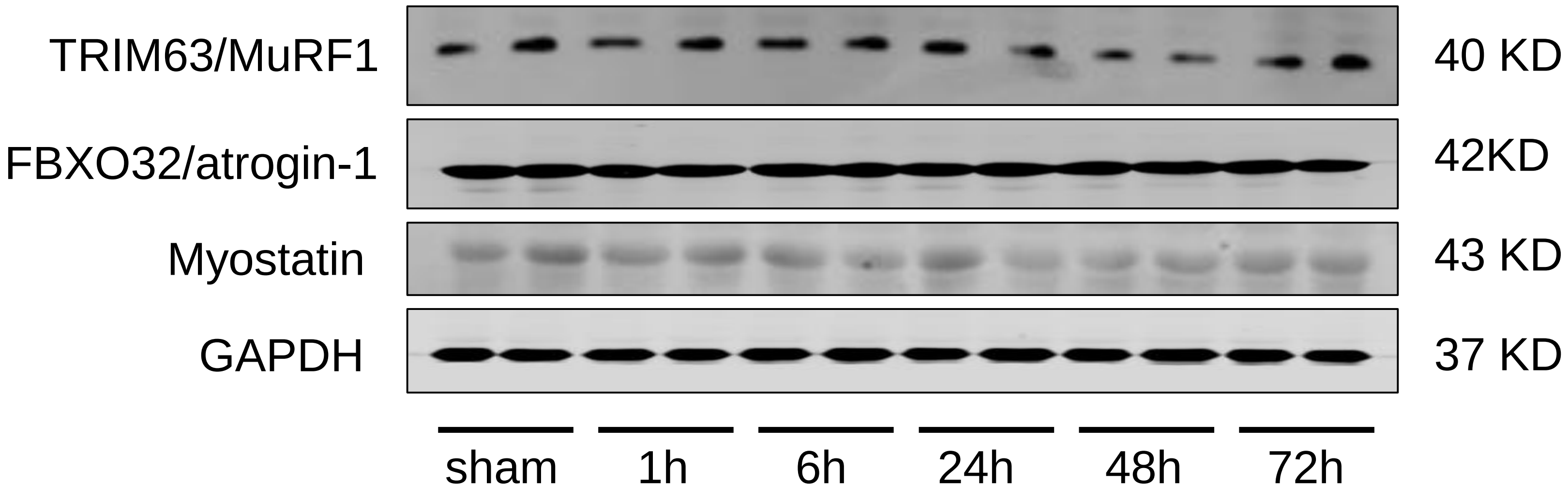

Supplementary Figure 1. Acupuncture does not change protein degradation markers in mice. Experiments were performed in the sham and Acu/LFES treated mice. Protein was isolated from the gastrocnemius muscle of mice immediately (0), 6-, 24-, 48- and 72-hours after Acu/LFES. The proteins TRIM63/MuRF1, FBXO32/atrogin-1 and myostatin were measured in skeletal muscle tissue lysates by Western blotting in sham and Acu/LFES mice.

supplementary Figure 2: Let-7 is decreased in the serum exosome- supplement

Supplementary Figure 2. Four members of Let-7 microRNA family are decreased in the serum exosome. Total RNA was isolated from serum exosomes of sham and Acu/LFES-treated mice. microRNA deep sequencing was performed, and the heat map showed that four let-7 microRNA carried by serum exosome are decreased by Acu/LFES. Left 3 lanes: sham levels; right 3 lanes: Acu/LFES mouse levels. Green indicates decrease; red indicates increase.

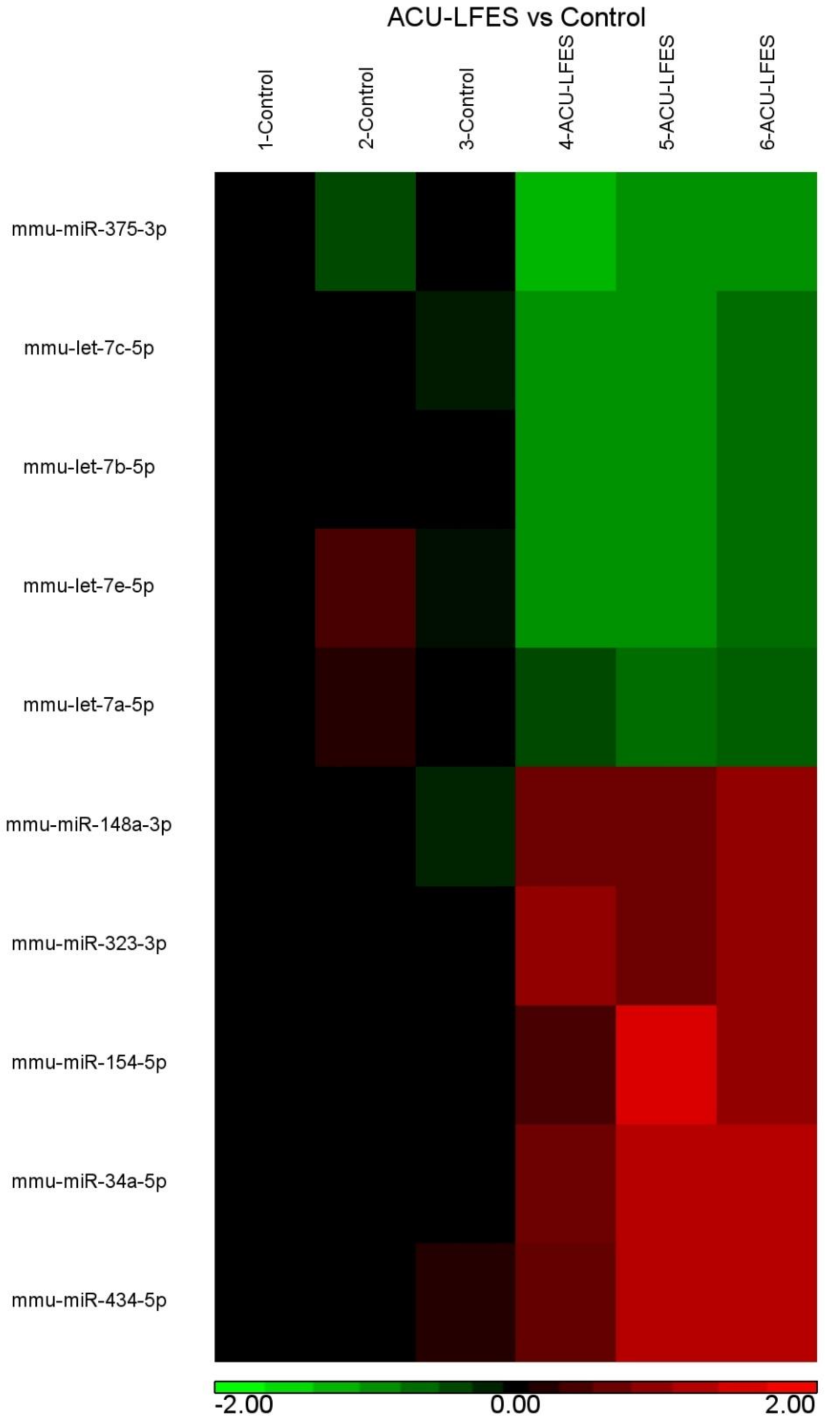

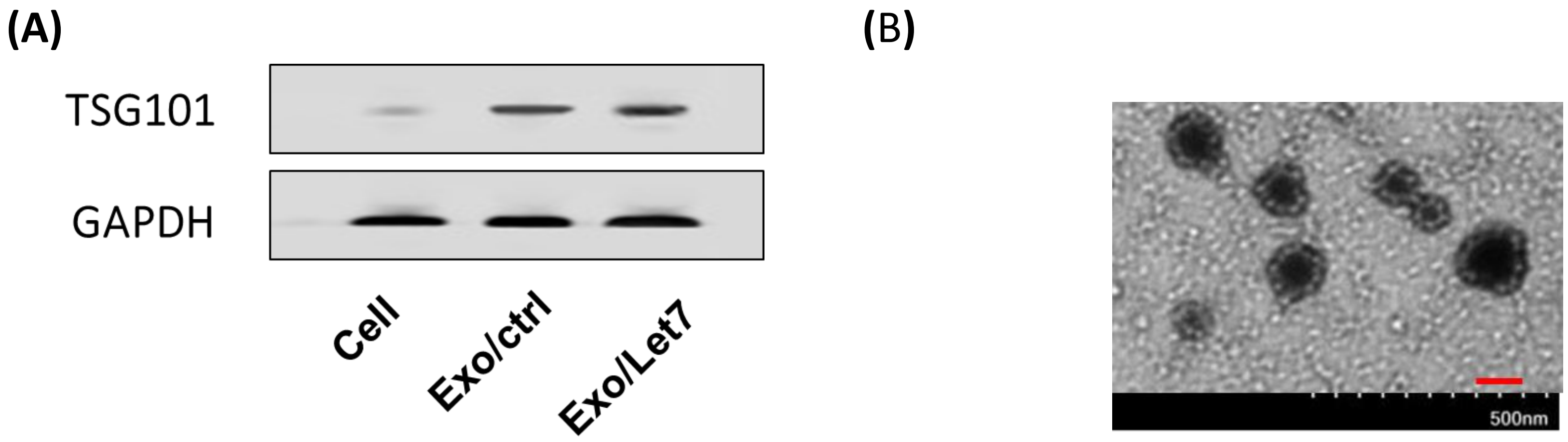

Supplement Figure 3: Exosome marker: the exosomes were harvested from culture conditional medium. The proteins from cultured cells and exosome were used to detect exosome maker protein, TSG101. The Western blot showed TSG101 abundance from cells, exosome with control microRNA mimic and with let-7 mimic. (B) Exosome imagine was taken by electro-microscope. The red scale bar indicate 100nm.

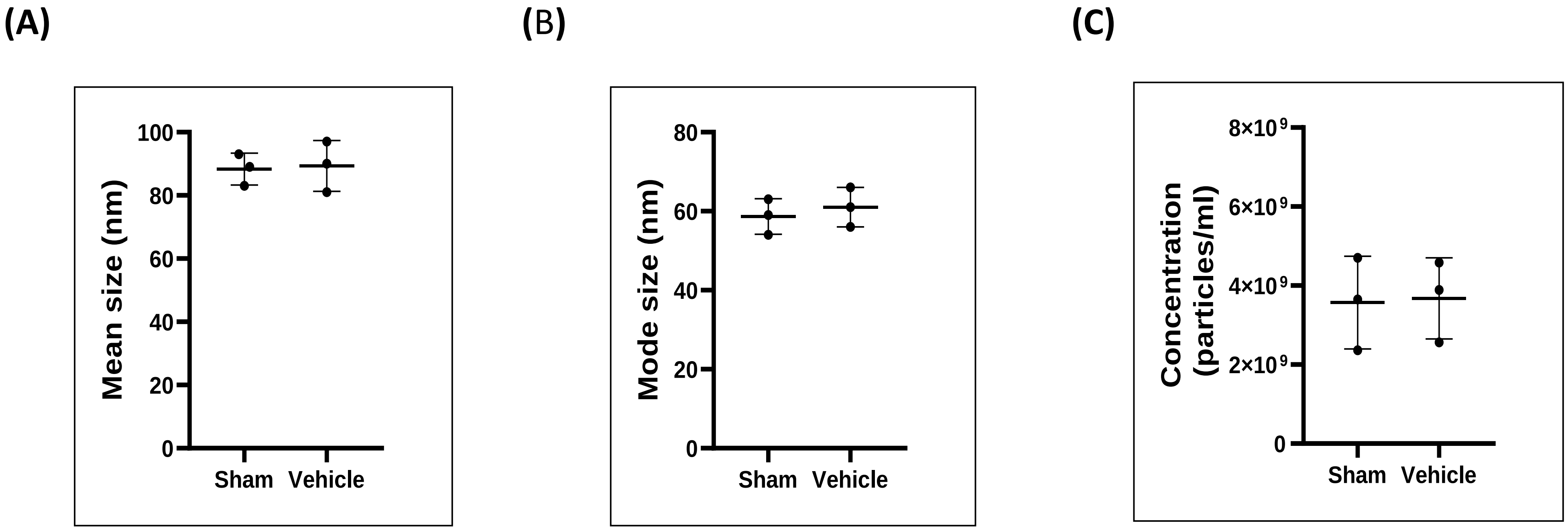

Supplementary Figure 4. Vehicle treatment does not change the exosome size and concentration compared with sham-injected mice. Exosomes were isolated from serum of mice with sham or vehicle treated. The exosome mean size (A), mode size (B) and concentration (C) were measured using a NanoSight instrument (means ± SE; n = 3/group).

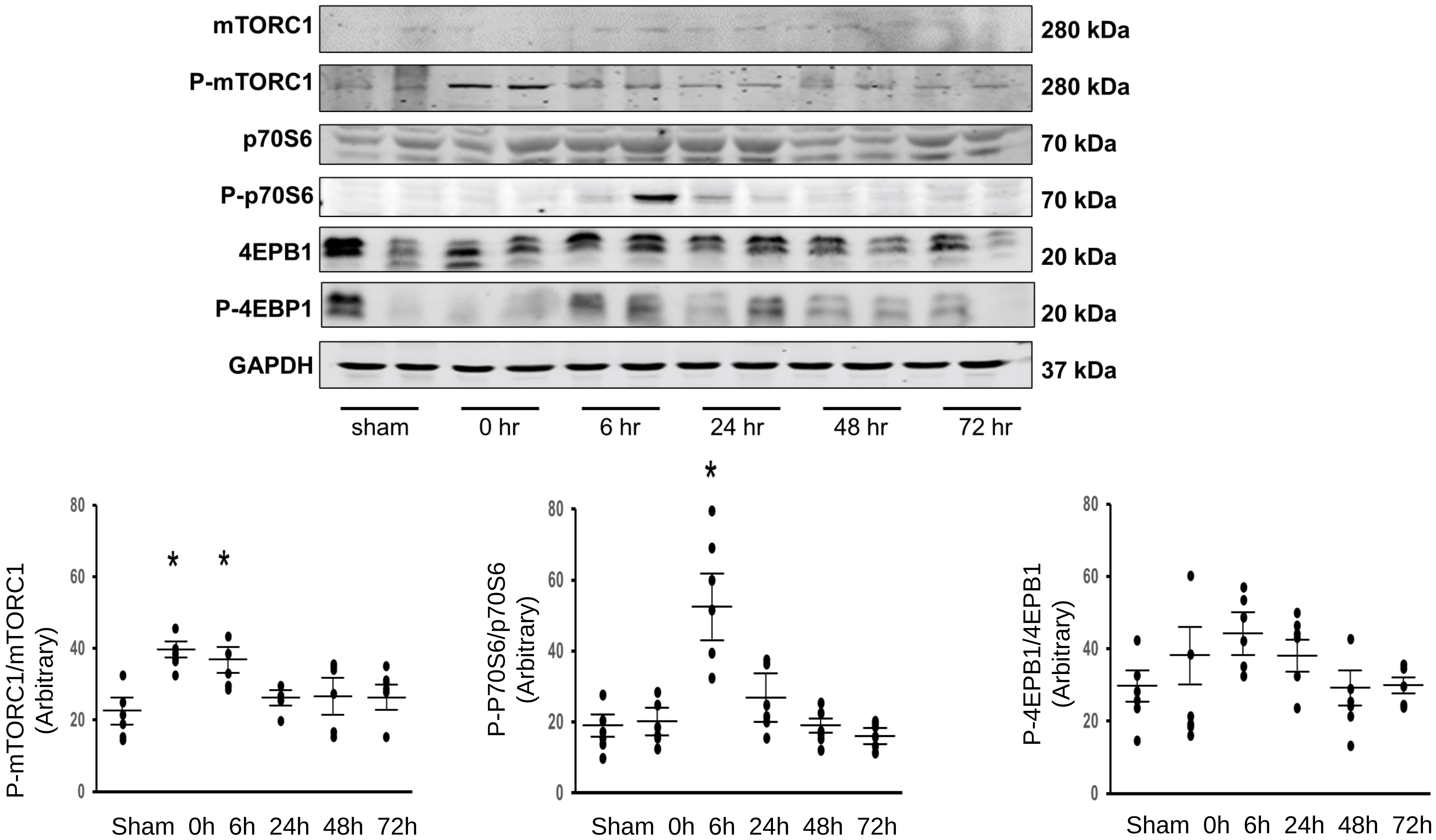

Supplementary Figure 5: Blocking exosome secretion limited the Acu/LFES-induced increase in protein synthesis in triceps brachii muscles.
